# Case Report: Two cases of immune-associated myocarditis caused by tislelizumab

**DOI:** 10.3389/fonc.2025.1549141

**Published:** 2025-11-03

**Authors:** Jie Zhao, Jinliang Wan, Shan Wang, Shuyue Zhang, Yanzhang Hao

**Affiliations:** Department of Oncology, Affiliated Hospital of Binzhou Medical College, Binzhou, China

**Keywords:** immune related myocarditis, tislelizumab, high dose methylprednisolone steroid therapy, case report, fatal myocarditis

## Abstract

The current phase of immune checkpoint inhibitors has improved the clinical outcomes of many cancer therapies; however, these agents may also lead to some high-consequence immune-related adverse events. We report two cases of cancer patients treated with tislelizumab, one with liver metastases after surgery for rectal cancer and one with non-small cell lung cancer. In both cases, elevated cardiac enzyme markers were observed, and one developed fatal myocarditis, while the other was treated with high-dose methylprednisolone steroid therapy and eventually improved. The probability of immune-associated myocarditis has been reported to be only 0.39%, but once myocarditis occurs, the mortality rate is more than 50%, making it a rare but serious side effect. Here, we discuss the antitumor treatment, the mechanism of immune-associated myocarditis induced by tislelizumab, the evaluation of the association, and the treatment after the development of immune-associated myocarditis in two patients, with the aim of improving clinical awareness of tislelizumab-induced immune-associated myocarditis and promoting timely judgment and appropriate therapeutic measures.

## Introduction

Cardiotoxicity can be a fatal complication associated with immunotherapy, with clinical manifestations ranging from asymptomatic elevated cardiac biomarkers to varying degrees of response such as heart failure, arrhythmias, cardiac fibrosis, cardiogenic shock, and fatal myocarditis (1, 2). Immune-associated myocarditis is a rare cardiotoxicity in immunotherapy with unknown predisposing factors. Although the reported incidence of immune-associated myocarditis is only 0.39%, it is often fulminant with a mortality rate of >50% (3). To date, there are few relevant cases of immune-associated myocarditis in the available reports. In this case study, we report a patient with non-small cell lung cancer and a patient with postoperative liver metastases from rectal cancer, who both developed immune-associated myocarditis during immunotherapy with tislelizumab. Clinical timeline and outcomes of two patients with tislelizumab-induced myocarditis (**Table 1**).

**Table 1:** Clinical timeline and outcomes of two patients with tislelizumab-induced myocarditis.

Patient	Age/Sex	Cancer type	Tislelizumab cycles	Symptom onset	Key Diagnostic findings	Treatment	Outcome
1	68/M	NSCLC	After 2nd cycle	Fatigue, flushing	↑CK, ↑TnI, LVEF 66%	Methylprednisolone (140mg → taper)	Improved
2	66/M	CRC with LM	After 2nd cycle	Low back pain, limb weakness	↑CK (15,084 U/L), ↑TnI (72.65 ng/mL)	Methylprednisolone 180mg, pyridostigmine bromide	Died

## Case presentation

### Patient 1

A 68-year-old male with no significant past medical history underwent a chest CT scan during a routine health examination over one year ago, which revealed a mass in the left lower lung lobe. Based on its imaging characteristics and clinical judgment, the lesion was preliminarily suspected to be malignant. Consequently, he underwent single port thoracoscopic left lower lobectomy with mediastinal and hilar lymph node dissection. Postoperative pathology confirmed the diagnosis of lung adenocarcinoma. Subsequently, he received multiple cycles of chemotherapy and radiotherapy. Following disease progression, third-line therapy was initiated with the anti-PD-1 antibody tislelizumab administered intravenously (200 mg every 3 weeks) via a peripherally inserted central catheter (PICC) line for three cycles. After completion of the second cycle, the patient developed fatigue and skin flushing, prompting hospital readmission. He was diagnosed with immune checkpoint inhibitor-associated myocarditis.

### Patient 2

A 66-year-old man, previously healthy, was diagnosed more than one year ago with liver metastases from colon cancer that were discovered during an examination. After receiving three cycles of targeted combination chemotherapy, he underwent left hemicolectomy with distal closure and transverse colostomy. Postoperatively, chemotherapy combined with immunotherapy was continued until disease progression, after which third-line treatment with tislelizumab combined with fruquintinib was initiated. Following two cycles of tislelizumab monoclonal antibody combined with fruquintinib, the patient developed back pain and bilateral lower limb weakness and was readmitted to the hospital, where he was diagnosed with immune-associated myocarditis. More details of the clinical presentation are shown in the table below (**Table 2**).

**Table 2:** More details of the clinical presentation are shown in thetable below:

Cases	1	2
Age	68 years	66 years old
Sex	Male	male
Vital signs on admission	T:36.1°C P:108 beats/minute R:25 beats/min BP:141/68mmHg	T:36.2°C P:103 beats/min R:25 beats/min BP:158/101mmHg
Pathologic type	Adenocarcinoma	Adenocarcinoma
Smoking status	Smoked for 30 years, 10 cigarettes/day, did not quit smoking	No
Gene mutations and immunohistochemistry	(Lower lobe of left lung) Mutations in exon 2 of KRAS gene, G12A/G12V/G12R/G12C/G13C. No mutations in EGFR/BRAF/NRAS/Her-2/PIK3CA/MET genes were detected. No fusions in ALK/ROS1/RET genes were detected. P40 (-), CK5/6 (-), CK7 (+), TTF-1 (+), NapsinA (-), and Ki-67 proliferation index of about 80%.	(Colon) No mutations were detected in KRAS/NRAS/BRAF/PIK3CA genes. ypT3N1cMx immunohistochemistry: msh2 (+)2; msh6 (+) mlh1 (+), pms2 (+) , HER2 (2+, indeterminate), Ki-67 positivity about 70%.
Comorbidities	None	Myasthenia gravis
TNM staging (AJCC 8th edition)	pT2aN1M0 Stage IIB	ypT3N1cM0 Stage IIIB
Pre-existing cardiac risk factors	None	None
Clinical symptoms at presentation	Weakness for more than half a month	Low back pain with bilateral lower extremity weakness for 3 days
Metastatic site	Lung metastasis	Liver metastases

### Patient 1 treatment and disease evolution

Upon admission, Patient 1 was diagnosed with adenocarcinoma of the left lower lung lobe (pT2aN1M0). Initial cardiac enzyme levels were elevated (**Table 3**), raising suspicion of immune checkpoint inhibitor-associated myocarditis. Echocardiography was subsequently performed, revealing a left ventricular ejection fraction (LVEF) of 66% with no significant structural cardiac abnormalities. Although the patient reported generalized fatigue, he denied chest pain or tightness. Based on the elevated cardiac enzymes, exclusion of alternative causes, and the clinical presentation, a preliminary diagnosis of immune checkpoint inhibitor-associated myocarditis was established, consistent with the diagnostic criteria for suspected ICI-myocarditis as per current guidelines (e.g., ESC/ASCO), which include: (1) temporal association with ICI administration, (2) elevated cardiac biomarkers, (3) exclusion of other common causes, and (4) supportive clinical features.

**Table 3:** Evolution of myocardial enzymes in Patient 1.

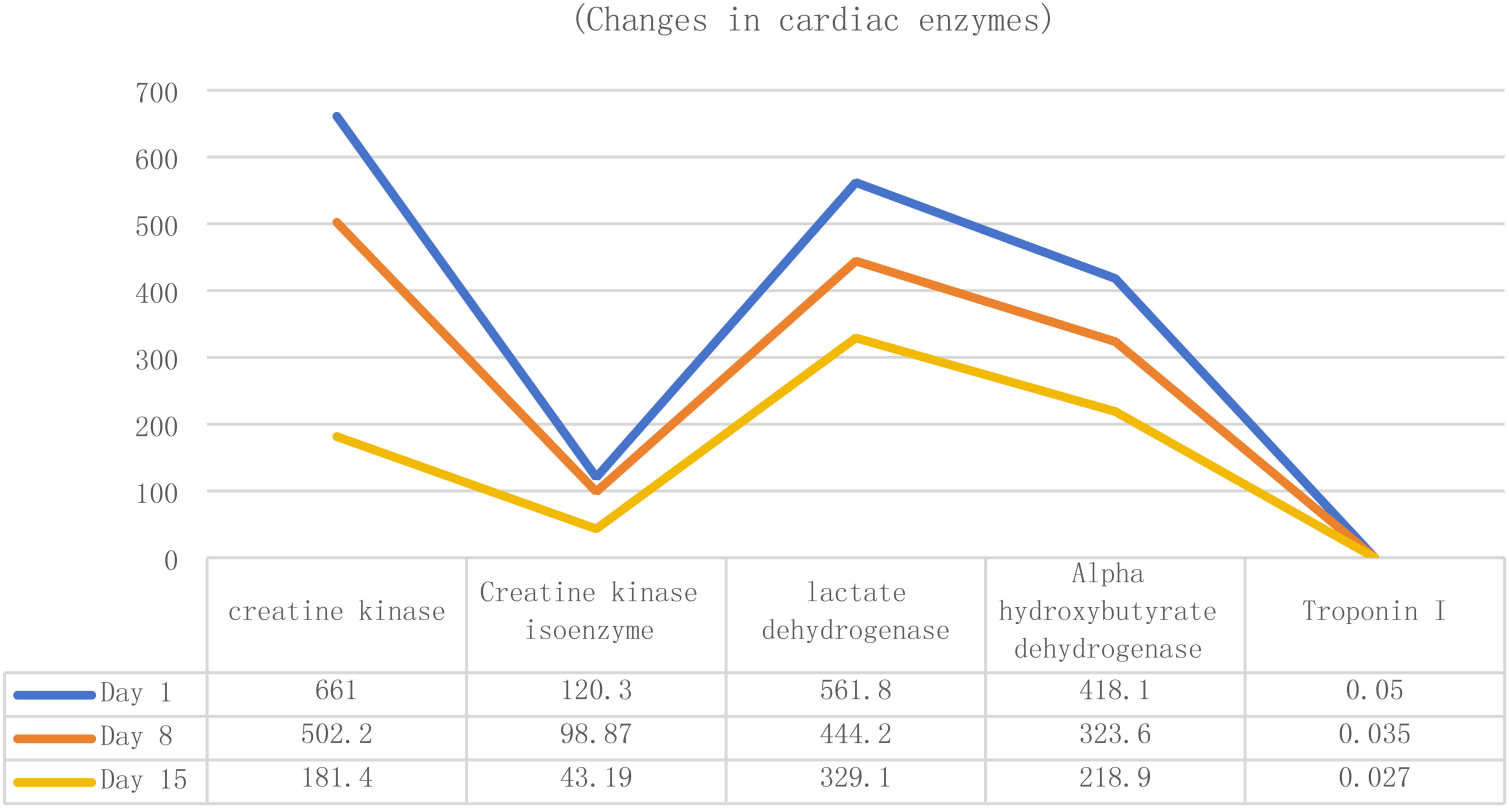

Therefore, the third cycle of tislelizumab treatment was stopped, and methylprednisolone sodium succinate 140 mg (once daily, IV) was administered immediately. After 3 days of treatment, the symptoms did not show significant improvement, indicating poor efficacy, so the dose was increased to 160 mg (once daily, IV). After 4 days, the symptoms improved significantly. Repeat myocardial examination and cardiac enzyme testing also showed improvement. The dose of methylprednisolone sodium succinate was then reduced to 80 mg (once daily, IV) for 5 days, then to 60 mg for another 3 days, and finally to 30 mg (once daily, IV). Subsequent review of cardiac enzyme levels and other indicators (**Table 3**) showed further improvement.

The patient’s symptoms improved markedly, and he was discharged from the hospital and transitioned to oral prednisone tablets 40 mg, with the dose reduced by 10 mg per week until discontinuation. The patient was followed up with regular chemotherapy without immunotherapy, and tumor control has remained stable to date.

### Patient 2 treatment and disease evolution

Admission diagnosis: colon cancer (ypT1cN1M0stage IIIB). Cardiac enzyme indexes at the time of consultation are shown in **Table 4** (4a-d, hereinafter referred to as **Table 4**). Cardiac enzyme indexes suggested that the heart damage was more severe, and cardiac ultrasound indicated a left ventricular ejection fraction of 58%. The patient complained of bilateral lower limb weakness and other discomforts but no chest pain or tightness. He was diagnosed with immune-related myocarditis. Therefore, the second cycle of tislelizumab treatment was stopped, and methylprednisolone sodium succinate 180 mg (once daily, IV) was administered.

**Table 4:** Evolution of myocardial enzymes in Patient 2.

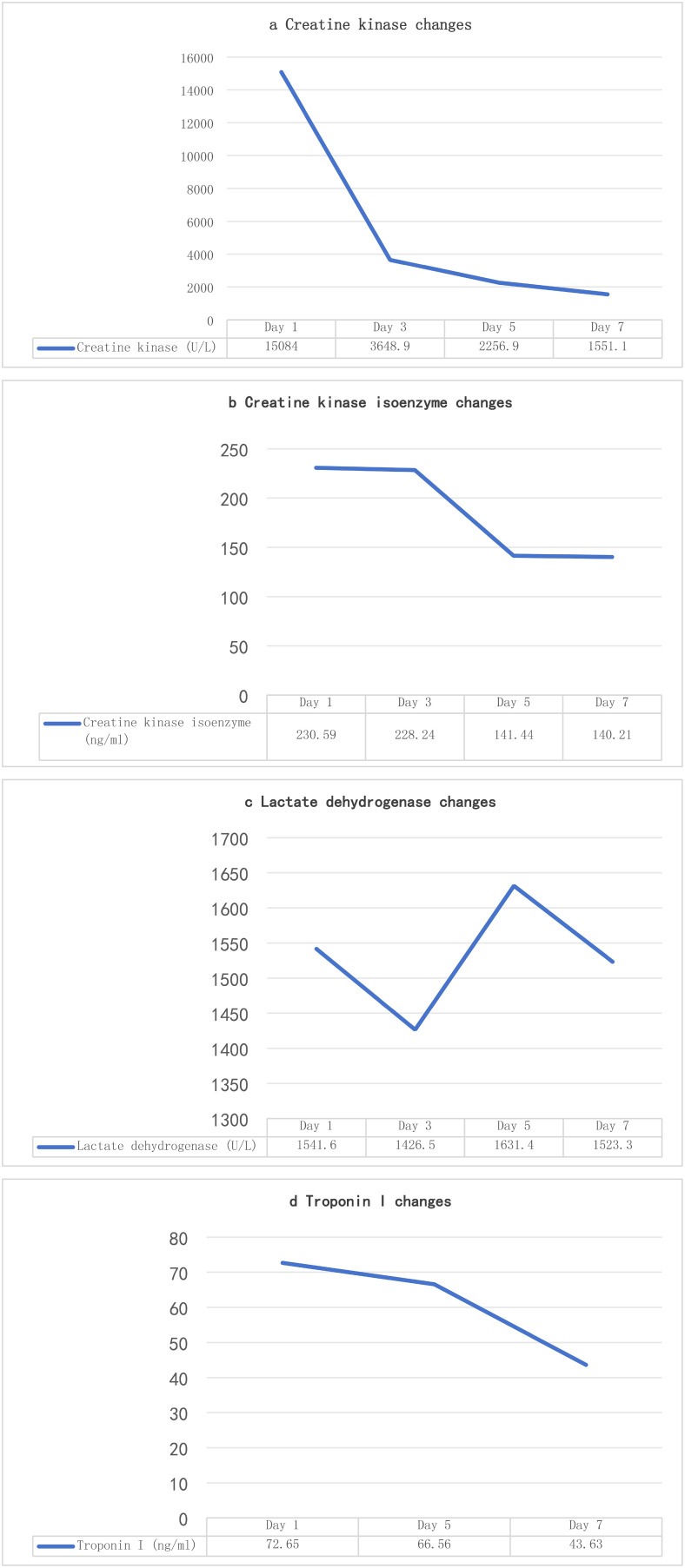

The patient’s symptoms did not show significant improvement. Considering the patient’s history of myasthenia gravis, cardiac enzymes (**Table 4**), immune indicators, and antinuclear antibody results (**Table 5**) were reviewed, suggesting that myocardial damage had worsened. As the hormone dosage had reached the maximum tolerated dose, the patient continued methylprednisolone sodium succinate 180 mg (once daily, IV) and was additionally treated with pyridostigmine bromide for myasthenia gravis. After 2 days, cardiac enzyme levels decreased (**Table 4**), and the patient’s symptoms slightly improved, although the condition remained serious. High-dose steroid therapy was continued, and repeat cardiac enzyme review after 2 more days (**Table 4**) showed further improvement. The patient’s cardiac enzyme levels and subjective symptoms were better than before.

**Table 5:** Immunological and antinuclear antibody parameters.

Name	Results	Reference value	Unit	Name	Result	Reference value	Unit
Antinuclear antibody (qualitative)	Negative(-)	Negative		(xMAP) anti-Scl-70 antibody	18	0-100	U/mL
Karyotype	.			(xMAP)anti-PM-Scl antibody	8	0-100	U/mL
Titer	.			(xMAP) anti-ribosome antibody	4	0-100	U/mL
(xMAP)anti-double-stranded DNA antibody	73	0–100 U/mL	U/mL	(xMAP) Anti-proliferating cell nuclear antigen antibody	26	0–100 U/mL (xMAP)	U/mL
(xMAP) Anti-SSB Antibody	5	0-100	U/mL	(xMAP)Anti-Nucleosome Antibody	2	0–100 U/mL	U/mL
(xMAP)Anti-Sm antibody	64	0–100 U/mL	U/mL	(xMAP)anti-histone antibody	4	0–100 U/mL	U/mL
(xMAP)anti-RNP antibody	15	0–100 U/mL	U/mL	(xMAP)Anti-filament B Antibody	3	0–100 U/mL	U/mL
(xMAP)Anti-Jo-1 Antibody	2	0–100 U/mL	U/mL	(xMAP) Anti-Mitochondrial Antibody M2 subtype	6	0–100 U/mL (xMAP)	U/mL
(xMAP) anti-SSA-60 antibody	10	0-100	U/mL	(xMAP)anti-SSA-52 antibody	5	0–100 U/mL	U/mL
Name	Result	Reference value	Unit	Name	Result	Reference value	Unit
Glutamate transaminase	425.10 ↑	15-40	U/L	Anti-chain O	30.85	0-200	IU/mL
Albuminous aminotransferase	201.80 ↑	9--50	U/L	Rheumatoid Factor	2.52	0--20	IU/mL
Glutamate/glutamine	2.11	0.3-3		C-reactive protein	24.40↑	0-6	mg/L
Total Protein	61.50 -	65-85	g/L	Creatine kinase	3648.90 ↑	25-200	U/L
Albumin	35.30	40-55	g/L	Creatine kinase isoenzyme	228.24 ↑	0--5	ng/ml
Globulin	26.20	20-40	g/L	Lactate dehydrogenase	1426.50 ↑	120--250	U/L
Albumin/Globulin	1.35	1.2-2.3		Immunoglobulin G	896.00	700--1600	mg/dL
Total bilirubin	7.57	5-23	μmol/L	Immunoglobulin A	225.60	70-500	mg/dL
Direct bilirubin	1.67	0-4	Immunoglobulin M	Immunoglobulin M	53.30	40-280	mg/dL
Indirect bilirubin	5.90	0-17	μmol/L	Complement C3	136.80	90-180	mg/dL
Alkaline phosphatase	169.50 ↑	45-125	U/L	Complement C4	28.80	10-40	mg/dL
Gamma glutamyl transpeptidase	95.50 1	10--60	U/L	Total Complement	60.64 ↑	23--56	U/mL

Therefore, the methylprednisolone sodium succinate dose was reduced to 120 mg (once daily, IV). However, two days later at 07:30, the patient’s symptoms suddenly worsened. Although alert, he exhibited altered mental status. Oxygen was immediately administered via nasal cannula at 6 L/min, achieving a peripheral oxygen saturation of 93%. Additional laboratory parameters are presented in **Tables 6a-d**. Electrocardiography demonstrated ventricular tachycardia. Considering an acute exacerbation of immune-related myocarditis, the patient was immediately transferred to the intensive care unit for continued treatment.

**Table 6:** Additional laboratory parameters.

A		
Parameter	Value	Reference Range
Vital Signs		
Temperature	36.6 °C	36.0-37.5 °C
Pulse	170 bpm	60–100 bpm
Respiratory Rate	21 breaths/min	12–20 breaths/min
Blood Pressure	110/74 mmHg	90-120/60–80 mmHg
B		
Physical Examination		
Skin Condition	Cold, clammy	
Pupils	Bilateral equal, round (3.0 mm diameter)	
Pupillary Light Reflex	Reactive	
Lung Auscultation	Clear breath sounds, no rales	
C		
Arterial Blood Gas (FiO_2_ 55%)		
pH	7.33	7.35-7.45
PaCO_2_	37 mmHg	35–45 mmHg
PaO_2_	72 mmHg	80–100 mmHg (room air)
Bicarbonate (HCO_3_^-^)	19.5 mmol/L	22–26 mmol/L
Base Excess (BE)	-6.4 mmol/L	-2 to +2 mmol/L
Oxygen Saturation (SaO_2_)	93%	>95%
D		
Laboratory Results		
Hemoglobin (Hb)	190 g/L	130–175 g/L
Sodium (Na^+^)	139 mmol/L	135–145 mmol/L
Potassium (K^+^)	4.6 mmol/L	3.5-5.0 mmol/L
Ionized Calcium (Ca^2+^)	1.02 mmol/L	1.12-1.32 mmol/L
Lactate (Lac)	7.0 mmol/L	0.5-1.6 mmol/L
Glucose (Glu)	18.8 mmol/L	3.9-6.1 mmol/L
Troponin I (TnI)	3.0 ng/mL	<0.04 ng/mL
NT-proBNP	8560 pg/mL	<125 pg/mL
Procalcitonin (PCT)	<0.12 ng/mL	<0.05 ng/mL
D-dimer	1.87 mg/L	<0.5 mg/L

At 13:15, electrocardiography showed ventricular tachycardia with a ventricular rate of up to 180 beats/min. Due to agitation, lidocaine (100 mg) was immediately administered as an intravenous bolus to control the ventricular rate, and dexmedetomidine was given for sedation. The ventricular rate gradually decreased, and the symptoms slightly improved.

At 13:32, ECG monitoring showed an escape heart rhythm, ventricular rate of about 90 beats/min, wide QRS complex deformity, and blood pressure of approximately 60/40 mmHg. The patient became unresponsive to verbal stimuli, pupils were dilated, and light reflexes disappeared. Endotracheal intubation and ventilator-assisted ventilation were immediately performed, along with continuous cardiopulmonary resuscitation. Norepinephrine and dopamine were continuously infused to raise blood pressure, and adrenaline was administered to enhance cardiac function, along with rapid fluid replacement and other supportive treatments.

At 14:00, the patient’s heart rate increased to 121 beats/min, with a ventricular rhythm, and invasive blood pressure rose to 71/63 mmHg. The patient remained in a persistent coma, continued on ventilator-assisted ventilation, and required large doses of vasoactive drugs to maintain blood pressure. At 15:04, cardiac monitoring showed a downward trend in heart rate and blood pressure, with the heart rate dropping to 60 beats/min. Cardiopulmonary resuscitation was immediately initiated. A 1 mg dose of epinephrine was given intravenously, followed by sodium bicarbonate for acid correction and potassium reduction. Despite active resuscitation efforts, heart rate and blood pressure did not recover. Cardiac monitoring showed no cardiac activity, and at 15:43, the patient was declared clinically dead.

## Discussion

### Mechanism of immune-related myocarditis triggered by tislelizumab

In contrast to the clear temporal pattern of classical chemotherapy cardiotoxicity, the onset and duration of immune-associated myocarditis are unpredictable, and the factors that predispose individuals to develop immune-associated myocarditis remain unclear (4). Studies (5) have suggested that patients with ICI-associated myocarditis exhibit high-frequency T-lymphocyte receptor sequences in cardiac, skeletal muscle, and tumor tissues, and that activated T-lymphocytes, in addition to attacking tumor cells, also non-selectively attack normal cardiac and skeletal muscle cells, triggering autoimmune myocarditis. Immune checkpoint inhibitors may also affect the cardiovascular system, resulting in cardiovascular adverse events. Similar studies have reported that immune-related myocarditis is a consequence of T-cell activation acting on both tumor cells and antigens common to cardiovascular tissues (6). The onset of immune-associated myocarditis is often associated with cardiac biomarker abnormalities and may also be accompanied by reduced ejection fraction, ventricular wall motion abnormalities, or other systemic injuries. Symptoms, signs, and laboratory tests are often nonspecific, making the diagnosis difficult. Our cases align with the typical presentation described in the literature, wherein symptoms often appear early in the treatment course. However, the divergent outcomes highlight the impact of patient-specific risk factors, such as a pre-existing autoimmune disorder (myasthenia gravis in Patient 2), which is recognized to potentiate the severity of ICI-related toxicity.

### Evaluation of the association of tislelizumab -induced immune myocarditis

**Figure 1:**
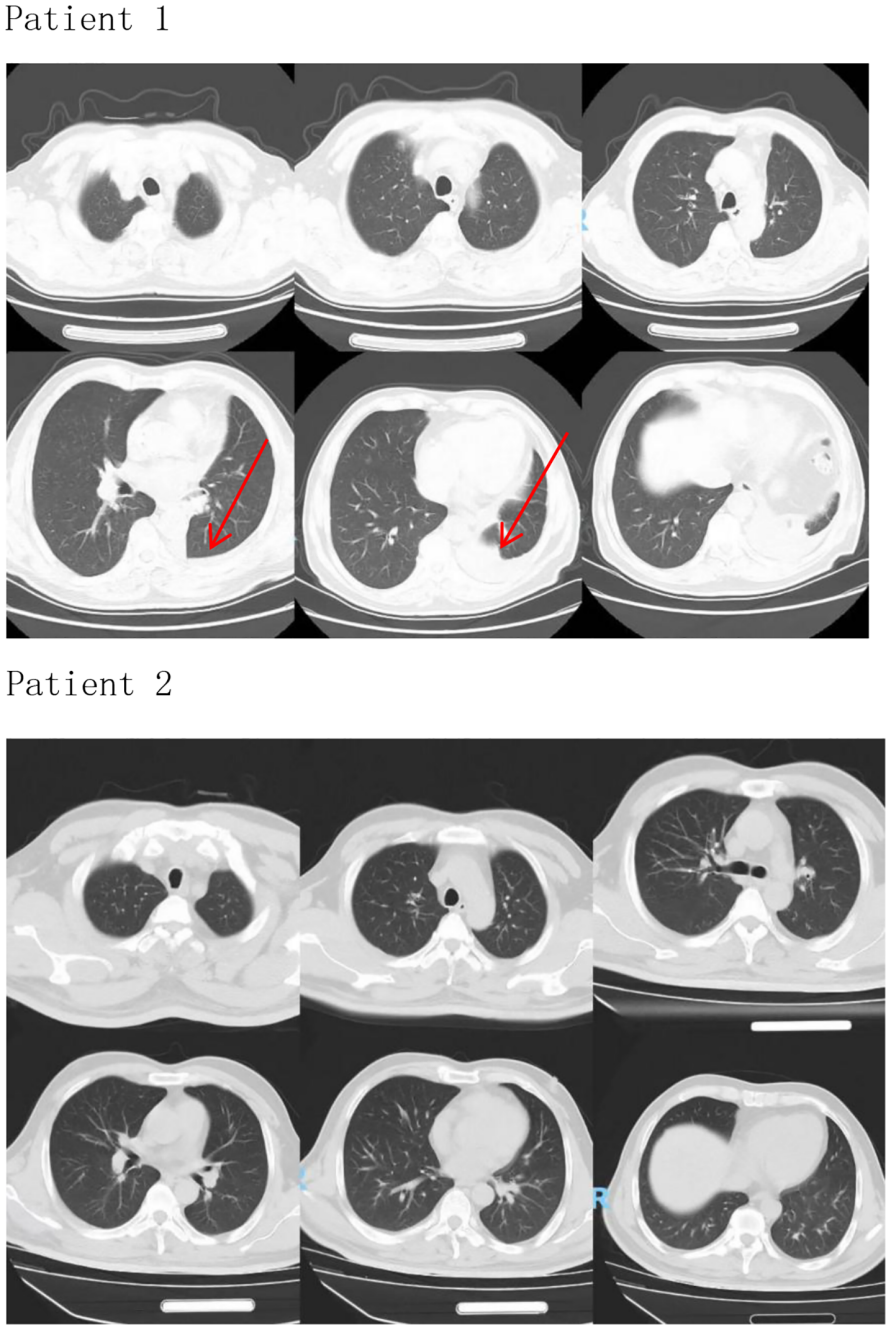
Patient 1 (The arrow indicates the left lower lobectomy site on the CT image.), Patient 2.

According to the association evaluation recommended by the National Center for Adverse Drug Reaction Monitoring:

1. Patient 1 developed flushing of the skin and fatigue at the end of the second course of treatment, and Patient 2 developed weakness of both lower limbs on the 18th day after the end of the first course of treatment, which is in line with reports (7). This is consistent with reports (7) that 64% of patients with immune-related adverse events received only one or two doses of tislelizumab before the onset of myocarditis, and there is a reasonable temporal relationship between tislelizumab and the development of myocarditis.
2. There are case reports of immune myocarditis caused by tislelizumab (8) According to VigiAccess, an adverse reaction database, the proportion of myocarditis, among all adverse reactions induced by tislelizumab, was 2.7%.
3. After stopping the drug, the corresponding treatment was given. Patient 1 improved, and myocardial enzymes gradually recovered. Although Patient 2 showed initial improvement in symptoms and cardiac enzyme levels, markers remained markedly elevated. Despite the preexisting history of myasthenia gravis (MG), we attribute the myocarditis to tislelizumab rather than MG for the following reasons::①Temporal Association: Symptoms manifested after the second cycle of tislelizumab (consistent with immune checkpoint inhibitor [ICI]-induced toxicity onset patterns), whereas MG had been stable for over a year prior to ICI initiation.②Pathophysiological Distinction: MG primarily involves antibody-mediated neuromuscular junction dysfunction (anti-AChR antibodies), with no established direct causal link to myocardial inflammation. ICI-induced myocarditis results from T-cell-mediated cytotoxicity against shared cardiac/neuromuscular antigens, amplified by PD-1/PD-L1 blockade.③Biomarker Profile: Marked elevation of cardiac-specific troponin (TnI) and NT-proBNP reflects acute myocardial injury, inconsistent with chronic MG pathology. Absence of MG exacerbation symptoms (e.g., ptosis, dysphagia) at myocarditis diagnosis further dissociates the events.
4. Literature Consensus: Current guidelines (NCCN, ESMO) recognize MG as a risk factor for ICI cardiotoxicity but not an independent cause of myocarditis. Fatal overlap syndromes (e.g., IM3OS) occur exclusively after ICI exposure in such patients. This aligns with Pathak et al.’s finding that pre-existing autoimmune conditions (like MG) potentiate ICI toxicity but require the inciting agent (tislelizumab) to trigger multi-organ autoimmunity. Severe myocardial injury resulted in a fatal outcome. Two patients were admitted to the hospital to check lung imaging, which did not show obvious abnormalities (**Figure 1**, Patient 1 and Patient 2). Only Patient 1 underwent left lower lobectomy. Infection indicators were normal, excluding intestinal infections, lung infections, lung cancer, and intestinal cancer as causes of fatigue and other symptoms. Therefore, the causal relationship between tislelizumab and immune-associated myocarditis in these patients is very likely.

### Combined use of drugs

Patient 1 was treated with a TP (paclitaxel + cisplatin) regimen for four cycles of chemotherapy followed by tislelizumab over a period of one year (9). A retrospective study (9) found that 77% of patients who developed cardiac adverse effects from cisplatin therapy exhibited acute cardiotoxicity. Chowdhury et al. (10) reported that 67% of patients treated with cisplatin had asymptomatic supraventricular tachycardia or ventricular tachycardia on monitoring, which resolved spontaneously after discontinuation of the drug but did not result in elevated cardiac enzymes. The patient did not experience elevated cardiac enzymes during chemotherapy with the TP regimen (**Table 7** displays cardiac enzyme profiles during four-cycle TP chemotherapy). A study (11) showed that cardiac adverse effects after the use of paclitaxel analogs mainly include arrhythmias, atrioventricular block, tachycardia, pericarditis, myocardial ischemia, and bundle branch block, but most of these adverse effects are self-limiting and recover spontaneously after discontinuing the drug. Of these, hypertension is the most commonly reported cardiotoxicity. The incidence of serious cardiotoxicity is low, and the incidence of grade 4 and 5 cardiotoxic events is about 0.5% in paclitaxel injection anticancer therapy, with the highest incidence of asymptomatic bradycardia and rarely elevated cardiac enzymes. In conclusion, the patient’s 2D echocardiography and cardiac enzyme results obtained before tislelizumab therapy were not abnormal, and there were no symptoms of malaise, making it unlikely that this outbreak of myocarditis was caused by the aforementioned drugs. Patient 2 also progressed after receiving cetuximab combined with oxaliplatin and fluorouracil for approximately one year, then progressed again after switching to bevacizumab combined with irinotecan and fluorouracil, and was subsequently switched to a third-line regimen of fruquintinib combined with tislelizumab. This combination has been associated with a significant increase in the incidence and risk of cardiovascular toxicity in patients with metastatic colorectal cancer. While fruquintinib is associated with cardiovascular toxicity (primarily hypertension and low-grade arrhythmias), its contribution to fulminant myocarditis in this case is unlikely for three reasons:①Divergent Toxicity Profiles: Fruquintinib-induced cardiotoxicity manifests as hypertension (19.2% incidence) and asymptomatic QT prolongation (≤3%) per phase III trials (12), contrasting with the observed T-cell-mediated myocarditis featuring CD8+ infiltration and troponin surge.②Temporal Dissociation: Myocarditis onset occurred after Cycle 2 of combination therapy, whereas fruquintinib monotherapy toxicity typically emerges within the first cycle (median 15 days; FALUCA study).③Biomarker Trajectory: Despite fruquintinib discontinuation during hospitalization, cardiac enzymes continued rising, indicating ongoing immune-mediated injury driven by tislelizumab’s PD-1 blockade rather than VEGF inhibition. Thus, while synergistic toxicity cannot be entirely excluded, the clinical, temporal, and pathophysiological evidence overwhelmingly implicates tislelizumab—not fruquintinib—as the principal driver of this fatal immune myocarditis (13).

**Table 7:** Cardiac enzyme profiles during four-cycle TP chemotherapy.

Cardiac enzyme	Reference range	After cycle 1	After cycle 2	After cycle 3	After cycle 4
Creatine Kinase (CK)	30–200 U/L	95	88	102	91
CK-MB	0–25 U/L	12	10	14	11
Troponin I (TnI)	<0.04 ng/mL	0.01	<0.01	0.02	0.01
Lactate Dehydrogenase (LDH)	120–250 U/L	175	168	190	182

In summary, in this case report, both patients were treated with tislelizumab, which has been proven to be safe and effective by large-scale clinical trials, and can be used in combination with chemotherapy for the first-line treatment of patients with advanced non-squamous NSCLC, with conditional approval of tislelizumab for the treatment of unresectable or metastatic microsatellite instability–high (MSI-H) or mismatch repair gene-deficient (dMMR) adult patients with advanced solid tumors, etc. Both patients received high-dose steroid therapy with starting doses of 140 mg/day and 180 mg/day, respectively. Tislelizumab was permanently discontinued after the occurrence of adverse reactions. Patient 1 showed improvement in symptoms after high-dose methylprednisolone steroid therapy, with a significant decrease in cardiac enzymes, and had no recurrence of disease during hormone use in the course of dosage reduction. For Patient 1, early detection and early diagnosis and treatment were timely. In contrast, Patient 2 suffered from a history of myasthenia gravis, which may have played a particularly critical role in the development of the disease process. Considering the patient was suspected of immune myocarditis, high-dose methylprednisolone steroid therapy was given immediately. However, the patient’s cardiac enzyme indexes continued to rise repeatedly, suggesting that the effect of high-dose methylprednisolone steroid therapy was still unsatisfactory. After the addition of brompisidomycin, cardiac enzyme indicators decreased. The patient was then given hormone reduction therapy; however, the disease worsened suddenly 2 days later, and he eventually died. Considering that the patient’s myocardium had already been greatly damaged when he was admitted to the hospital, and that although myasthenia gravis was a high-risk factor for his death, review of immune and antinuclear antibody indexes did not show any obvious abnormalities, it was considered that the patient’s immune-associated cardiomyopathy triggered by tislelizumab was more severe, resulting in an extremely serious adverse event. It is suggested that early diagnosis and judgment of the severity of the disease appear to be important in clinical work when dealing with such events related to serious adverse reactions. In comparison to previously reported cases of tislelizumab-associated cardiotoxicity, our report underscores that the presence of a pre-existing neuromuscular autoimmune disease (myasthenia gravis) may signify a high-risk subgroup for developing severe and fatal myocarditis, necessitating extreme vigilance.

## Summary

The stark contrast in outcomes between these two patients emphasizes the critical importance of risk stratification prior to initiating ICIs like tislelizumab. A thorough assessment to determine of a history of autoimmune diseases is mandatory. The two patients exhibited varying degrees of cardiac injury with divergent outcomes, underscoring the necessity for proactive and regular cardiac function assessment during tislelizumab therapy, including baseline and serial measurements of troponin and brain natriuretic peptide (BNP) levels, as well as electrocardiograms, especially during the first 6 weeks of treatment, to enable early detection and diagnosis of myocardial damage. Upon confirmation of immune myocarditis, immediate discontinuation of tislelizumab and initiation of high-dose methylprednisolone steroid therapy are imperative. As highlighted by a systematic review of 60 patients with immune checkpoint inhibitor (ICI)-induced myocarditis/myositis/myasthenia gravis overlap syndrome (IM3OS), this condition carries high lethality. Key findings include median symptom onset after the first ICI dose, predominant manifestations of fatigue (80%) and muscle weakness (78%), markedly elevated creatine kinase (mean: 9,645 IU/L), and arrhythmias in 67% of patients. Despite universal high-dose steroid administration, in-hospital mortality reached 60%, with no survivors rechallenged with ICIs (14). This syndrome reflects fatal multisystem autoimmune involvement, necessitating proactive neuromuscular toxicity screening in ICI-related myocarditis cases (and vice versa), where early multidisciplinary intervention may improve outcomes. Integrating these clinical data with our severe adverse events, we emphasize that timely and adequate steroid therapy must be administered, with dose reduction considered only after documented improvement in cardiac enzymes. Particular caution in steroid tapering is warranted for patients with underlying high-risk factors. These cases further provide therapeutic reference for managing tislelizumab-induced immune myocarditis.

## Data Availability

The original contributions presented in the study are included in the article/**Supplementary Material**. Further inquiries can be directed to the corresponding author.
